# Investigating the effectiveness of the Mediterranean diet in pregnant women for the primary prevention of asthma and allergy in high-risk infants: protocol for a pilot randomised controlled trial

**DOI:** 10.1186/1745-6215-14-173

**Published:** 2013-06-14

**Authors:** Dean A Sewell, Victoria S Hammersley, Graham Devereux, Ann Robertson, Andrew Stoddart, Chris Weir, Allison Worth, Aziz Sheikh

**Affiliations:** 1 School of Life Sciences, Heriot-Watt University, Riccarton, Edinburgh EH14 4AS, UK; 2 Public Health Nutrition Research Group, University of Aberdeen, Aberdeen AB25 2ZD, UK; 3 Centre for Population Health Sciences, The University of Edinburgh, Medical School, Teviot Place, Edinburgh EH8 9AG, UK; 4 Edinburgh Health Services Research Unit/Edinburgh Clinical Trials Unit, Western General Hospital, Edinburgh EH4 2XU, UK; 5 MRC Hub for Trials Methodology Research, Centre for Population Health Sciences, The University of Edinburgh, Medical School, Teviot Place, Edinburgh EH8 9AG, UK; 6 Allergy and Respiratory Research Group, Centre for Population Health Sciences, The University of Edinburgh, Medical School, Teviot Place, Edinburgh EH8 9AG, UK

**Keywords:** Allergy, Asthma, Mediterranean diet, Pregnancy

## Abstract

**Background:**

Over recent decades there has been a substantial increase in asthma and allergic disease especially in children. Given the high prevalence, and the associated high disease burden and costs, there is a need to identify effective strategies for the primary prevention of asthma and allergy. A recent systematic review of the literature found strong supportive epidemiological evidence for a protective role of the Mediterranean diet, which now needs to be confirmed through formal experimental studies. This pilot trial in pregnant women aims to establish recruitment, retention and acceptability of a dietary intervention, and to assess the likely impact of the intervention on adherence to a Mediterranean diet during pregnancy.

**Methods/Design:**

This study was a pilot, two-arm, randomised controlled trial in a sample population of pregnant women at high risk of having a child who will develop asthma or allergic disease.

**Discussion:**

The work ultimately aims to contribute to improving health outcomes through seeking to reduce the incidence of asthma and allergic problems. This pilot trial will prove invaluable in informing the subsequent planned large-scale, parallel group, randomised controlled trial.

**Trial registration:**

ClinicalTrials.gov: NCT01634516

## Background

In recent decades, there has been a marked worldwide increase in asthma and allergic disease prevalence, especially in children [1,2]. Children bear the greatest burden of asthma and allergic disease, principally atopic dermatitis/eczema, allergic rhino-conjunctivitis and food allergy [3]. Asthma is a major public health concern – the World Health Organization estimates that globally 235 million people currently have asthma and that it is now one of the most common childhood diseases requiring chronic drug therapy [4].

Estimates state that in the UK 1.1 million children and 4.1 million adults receive treatment for asthma. The cost of asthma for the UK economy was estimated to be £2.3 billion in 2001, comprising £1.2 billion in lost productivity and £889 million in National Health Service (NHS) expenditure [5,6]. The UK prevalence of atopic dermatitis/eczema and hay fever in children is also high, such that internationally the UK was ranked second for prevalence of atopic dermatitis/eczema and seventh for hay fever [7,8]. In Scotland in 2004, nearly 4% of general practitioner consultations and 1.5% of hospital admissions were for allergic disorders [9].

There is increasing evidence suggesting that prenatal and early-life exposures influence the development of asthma and allergic diseases, with interest converging on the possible role of the diet during pregnancy and early life. It has been hypothesised that the maternal diet during pregnancy modulates the development of asthma and allergic disease by influencing foetal airway and/or immune development [10]. Birth cohort studies have reported associations between aspects of maternal diet during pregnancy and childhood asthma and allergic outcomes [11]. A recent systematic review and meta-analysis examined the strength of the scientific evidence for associations between dietary intake of foods and nutrients by pregnant women and children, and the risk of children developing asthma and other allergic disorders [12]. Of nearly 15,000 potentially relevant articles, 62 satisfied the inclusion criteria. There were no randomised controlled trials (RCTs); all were cohort, case–control or cross-sectional studies. Although these study designs are potentially at high risk of bias in assessing the effectiveness of dietary constituents/patterns, the review results found a potentially substantial protective role for vitamins A, D and E, zinc, fruit and vegetables, and a Mediterranean diet (MD) for the prevention of asthma and allergic disorders. Of these dietary candidates, vitamin D [13] and vitamin E [14] are under investigation. In general, the MD is characterised by the elevated intake of fruits and vegetables, olive oil, legumes, nuts and fish [15]. The study of dietary patterns represents a broader picture of food and nutrient consumption compared with single food items and nutrients [16,17].

Of the five observational MD studies in the review, one used a cohort design [16] and four were cross-sectional studies [18-21]. The higher quality cohort study reviewed by Nurmatov and colleagues [12] reported that high adherence to a MD was found to be protective against persistent wheeze (odds ratio = 0.22, 95% confidence interval = 0.08, 0.58), atopic wheeze (odds ratio = 0.30, 95% confidence interval = 0.10, 0.90) and atopy (odds ratio = 0.55, 95% confidence interval = 0.31, 0.97) [16]. There are large geographical differences in the prevalence of asthma, with higher prevalence rates in English-speaking countries and lower prevalence rates in, for example, the Mediterranean region [22,23]. As a result of a high intake of olive oil, vegetables and fruit, wholegrain cereals and bread, a low intake of dairy products, some fish consumption, and only small amounts of red meat, a MD is rich in antioxidants but also high in carbohydrates, fibre and unsaturated fatty acids and low in saturated fatty acids.

Whilst observational studies have reported potentially beneficial associations with a MD, it is not known whether an intervention to promote the MD reduces the likelihood of childhood asthma and allergic disease. There are currently no RCTs testing the hypothesis that a MD decreases the risk of asthma and allergic disease in children. Before testing this hypothesis in a large-scale RCT, pilot work is needed to ensure there is a robust measure to assess the MD and to investigate the feasibility of achieving and sustaining a dietary change. We are carrying out a pilot trial of an intervention to promote a MD, to inform a subsequent multi-centre RCT. We report here the protocol for the pilot trial in a sample of pregnant women.

### Aims of the study

The primary aims of the pilot trial are to investigate rates of maternal recruitment and retention in the control and intervention groups, and to assess the acceptability of dietary advice and dietary modifications in the intervention group.

The secondary aims are to determine whether there are measurable changes in the MD score in both groups, and to measure adherence to a MD during the last trimester of pregnancy in the intervention group.

### Specific research questions

The research questions are as follows: What are the recruitment and retention rates of pregnant mothers whose infants are at high risk of asthma/allergy? To what extent do mothers adhere to a MD? Can a MD score be increased in pregnancy? Can any increases in MD adherence be sustained during pregnancy? Can a measurable change in a biomarker of oxidative stress be detected as a result of adherence to a MD? Is the advice and dietary modification acceptable to participants? To inform cost-effectiveness considerations, can a breakdown of intervention delivery time (initial contact and number and duration of follow-up telephone calls) be measured?

## Methods/Design

### Ethical and research and development approval

A favourable ethical opinion was obtained from the NHS Lothian South East Scotland Research Ethics Committee 02 on 12 April 2012 (REC reference 12/SS/0052). NHS Lothian R&D Management Approval was obtained on 27 April 2012 (R&D No. 2012/SJ/DN/01).

### Trial design

We are carrying out a two-arm, pilot, parallel-group RCT. Trial participants are being recruited from two maternity service sites in Scotland, both with dating scan appointment rates of approximately 100 per month. Eligible participants receive either: diet advice and support, with a supporting MD resource booklet, in addition to standard care; or standard care with no additional dietary advice/support/materials.

### Eligibility of participants entering the trial

#### Inclusion criteria

The inclusion criteria are as follows: pregnant in first trimester; age ≥16 years; a history of atopic dermatitis/eczema, food allergy, allergic rhinitis (persistent or intermittent) or asthma in the pregnant woman, her partner, or children; and willing to give informed consent.

#### Exclusion criteria

The exclusion criteria are as follows: not pregnant; age <16 years; no history of atopic dermatitis/eczema, food allergy, allergic rhinitis (persistent or intermittent) or asthma in the pregnant woman, her partner, or children; recent (within the last 3 months) or current involvement in a dietary or supplementation trial; and unable/unwilling to give informed consent.

### Recruitment

#### Hospital/community treatment centre

We have recruited one hospital and one community treatment centre in the same NHS Scotland Board/region that carry out dating scans.

#### Participants

A letter of invitation to join the pilot trial, information for participants and a consent form are being sent out to women along with their dating ultrasound scan appointment letter and pack. These are sent by a health records department running a centralised regional telephone booking system and booking clerks are keeping a record of invitations sent. Interested participants are invited to contact the researcher by text, telephone or email to discuss the trial. They are then screened for eligibility to take part in the trial. Eligibility of women at high asthma/allergic risk for the foetus is based on a positive answer to the question: ‘Do you (the mother), or the father, or sibling of the baby have an allergic disease: eczema, a food allergy, hay fever or asthma?’ Details are recorded on the screening questionnaire.

Enrolled participants are sent a food frequency questionnaire (FFQ; Scottish Collaborative Group Food Frequency Questionnaire version 6.6) for completion at home and a small urine collection vessel with instructions to fill the container on the day of the scan and bring that, the consent form and the completed FFQ to their ultrasound scan appointment. The researcher then arranges to meet the participant in the clinic waiting area after their dating scan, where the researcher collects the consent form, urine sample and FFQ and administers a baseline MD questionnaire (based on [18]). Urine specimens are being stored frozen at -80°C for subsequent analysis.

There are two further opportunities for recruitment. First, following invitation, when nonresponders arrive in the clinic they may see a poster in the clinic advertising the study. If they would like to take part they can ask the clinic staff to point out the researcher to them. If the potential participant has already read the information for participants then they are able to consent and can be recruited after the dating scan that day. Second, those who are interested, but have not read the information for participants are given a further period of time (that is, ≥24 hours) before being contacted by the researcher to see whether they wish to participate; if recruited and randomised to the intervention group, the participant is asked to return to the hospital for the intervention or alternatively the researcher can arrange a home visit.

Consent is obtained to inform the participants’ general practitioner of her participation in the trial, and a letter is subsequently sent to the general practitioner.

### Randomisation

Participating women are randomised when visiting the hospital for their dating ultrasound scan. Allocation to the intervention arm or control arm is by restricted randomisation (by site), via pre-randomised sealed envelopes, based on a predetermined random number allocation. This randomisation has been carried out by an independent statistician. Participants are randomised after consent has been obtained and immediately after the first MD questionnaire has been completed. Participants are enrolled for a total of ~6 months (that is, from 12 to 36 weeks of pregnancy).

### Intervention

The intervention is a 15-minute structured dietary advice session encouraging the consumption of particular foods that are consistent with the MD, developed with a dietitian and administered by a researcher or hospital dietitian using a booklet. The initial session is directed at increasing consumption of MD foods, with subsequent supportive telephone calls from the dietitian/researcher at 4, 8 and 18 weeks post enrolment, to review and revise MD goals. The booklet contains text and pictures, with frequently asked questions and answers and ideas for modifying the diet, such as eating more fruit, vegetables, using olive oil and eating more fish. Advice for cutting back on fat on meat is also included. A MD pyramid (adapted from [24]) is contained in the booklet and discussed. The dietitian/researcher also discusses ideas for participants to reach the goals of eating more fruit, vegetables and fish in the context of current portion consumption, and reducing red and processed meat by exchanging with white meat. The use of olive oil for cooking and dressings is encouraged, and participants are given a shopping voucher (£10) at baseline and 12 weeks post baseline that can be used for purchasing olive oil. Olive oil used for cooking and dressings is an important component of the MD. The MD booklet also has recipes for a soup, a fresh sauce and a vegetable curry, all of which have olive oil as an ingredient. No energy restrictions are suggested, and the target of at least five portions of fruit and vegetables per day is emphasised.

#### Intervention arm

After the dating scan the researcher collects the consent form, the FFQ and the urine sample and checks that the participant has previously received standard dietary advice for pregnant women, including the importance of folic acid and vitamin D supplementation. Participants then receive a 15-minute dietary advice session. A supermarket voucher to the value of £10 is provided.

At 12 weeks post-baseline, a second MD questionnaire with a reply envelope for completion and return is posted to the participant and followed up by a telephone call from the researcher. A further supermarket voucher to the value of £10 is sent to the participant on receipt of the completed questionnaire, accompanied by advice to use it to buy olive oil. Non-returns of diet questionnaires are followed up by a telephone enquiry from the researcher after 1 week, with a reminder letter and another questionnaire being sent out after 2 weeks without a response.

Two weeks before the 24 weeks post-baseline MD questionnaire, participants are contacted by telephone to arrange a home visit and are sent a second FFQ. When visited at home by the researcher, a third MD questionnaire and second FFQ and a urine sample are collected.

Free telephone access to the researcher is available throughout the study; the frequency and duration of calls to the researcher are being recorded.

#### Control arm

After the dating scan the researcher collects the consent form, the FFQ and the urine sample and checks that the participant has previously received standard dietary advice for pregnant women, including the importance of folic acid and vitamin D supplementation. These participants do not receive the 15-minute dietary advice session. A supermarket voucher to the value of £10 is provided. The first follow-up by the researcher is by telephone 12 weeks post-baseline to inform them they will receive the second MD questionnaire and reply envelope for completion and return. A further supermarket voucher to the value of £10 is sent to the participant on receipt of the completed questionnaire. Non-returns of diet questionnaires are followed up by a telephone enquiry from the researcher after 1 week, with a reminder letter and another copy of the questionnaire being sent out after 2 weeks without a response.

Two weeks before the 24 weeks post-baseline MD questionnaire, participants are contacted by telephone to arrange a home visit and are sent a second FFQ. When visited at home by the researcher, a third MD questionnaire and second FFQ and a urine sample are collected.

### Assessment of outcomes

#### Recruitment and retention

The number of women who received an invitation, responded, were eligible, were recruited and completed the trial will be recorded.

#### Measuring the Mediterranean diet score

The MD score is being measured at baseline (around 12 weeks of pregnancy) and at 12 and 24 weeks post baseline MD questionnaire (i.e. approximately weeks 24 and 36 of pregnancy). The number of times participants consume particular food groups in the last week is classified into never, one or two times, or three or more times. For beneficial components (for example, vegetables, legumes, fruits, cereals, fish, dairy products) the frequency scoring is higher than for components considered less beneficial (meat, fast food, confectionery). As our trial involves pregnant women, we assume dairy products to be protective and we will not include alcohol consumption in the score [16]. This is because of the increased requirement for calcium and the fact that alcohol consumption is not recommended in pregnancy. A 3-point increase in MD score on a scale of 22 could equate to potentially moving from a low-quality maternal MD score into the optimal range of MD score based on the study of Chatzi and colleagues [16].

#### Biomarker analysis

Urine samples are being collected from participants at baseline and 24 weeks post intervention. We will measure levels of urinary 8-hydroxy-2′-deoxyguanosine, the ratio of nitrite to nitrate, and ferric-reducing antioxidant power. Urinary 8-hydroxy-2′-deoxyguanosine is a sensitive, stable and integral marker of oxidative stress *in vivo*[25]. The stable metabolic products of nitrous oxide and nitrite/nitrate are markers of whole-body nitrous oxide production. Total antioxidant activity is measured by the ferric-reducing antioxidant power. These analytes will be used as outcome measures in order to relate changes in oxidative stress, activity and nitrous oxide production to changes in MD score.

#### Food frequency questionnaire

FFQ estimates of nutrient intake will be used to support the MD score and to compare the intervention and control groups.

#### Qualitative evaluation of the trial

A sample of participants will be contacted by telephone at the end of the trial period for a recorded semi-structured telephone interview by one of the researchers not involved in meeting the participants. The sample will be weighted 2:1 in favour of the intervention group. This interview will address participants’ views regarding the acceptability of the intervention, any concerns and suggestions to improve the trial.

#### Health economics

We are recording the time taken to deliver the intervention, and the frequency and duration of follow-up telephone calls, to assess the feasibility of reporting intervention costs in a subsequent large-scale RCT. Any problems arising in recording this information will be reported. The patterns of researcher/dietitian time usage in the pilot trial will be used to inform the design of the proposed subsequent RCT.

#### Measurement of potential confounders

We are collecting data related to potential confounders such as the baby’s birth weight, sex, socioeconomic status, maternal education level and exposure to smoking during pregnancy. Given the small sample size in this pilot trial, there will be a limit to how many confounders it will be possible to adjust for in the analysis; however, we will be able to describe their distribution in the two groups.

### Sample size

Our intention is to continue recruitment until we have 50 women included in the trial in order to answer our research questions. Both centres have approximately 100 dating scans per month, but there is seasonal variation. We estimate that at least 800 invitations will be made, and those who do not respond prior to the ultrasound scan appointment have a further opportunity to take part if they respond to the clinic poster. Of the 800+ invitations made, we estimate that one-quarter (*n* = 200) will fulfil the eligibility criteria (‘Do you (the mother), or the father, or sibling of the baby have an allergic disease …?’). This estimate is based on an overall assessment of the epidemiology of allergic disorders in Scotland, systematic reviews of a number of previous primary prevention trials [26,27] and a recent dietary intervention trial in pregnant women [14]. Based on our discussions with consumer representatives that have informed this pilot trial, we anticipate that ~25% (that is, *n* = 50) of eligible women will be willing to take part and, with an estimated 20% attrition/incomplete data collection during the course of the pregnancy, we anticipate that 40 participants will complete the study. If these assumptions are fulfilled, recruitment and retention will be comparable with other leading international primary prevention trials [27].

As this is a pilot trial, using a questionnaire based on Castro-Rodriguez and colleagues [18], we estimate that using a level of 0.05 (two-tailed) and power of 80% will require a sample size of 40 to detect a statistically significant difference in the mean MD score of the intervention group, from baseline, in the order of 3 units.

### Statistical analysis plan

Data analysis, using the following analysis plan, will be blind to the allocation arm.

#### Descriptive analysis

For each treatment arm we will describe the participant age (mean and standard deviation), birth weight, gender of baby, number of previous children, the eligibility qualifying criteria, and socioeconomic factors.

#### Recruitment and retention

Recruitment rate will be calculated as the proportion of women invited to take part compared with the number of women: responding to the invitation; meeting the eligibility criteria; and recruited into the trial.

The retention rate will be calculated as the proportion of women starting the study to those completing to 24 weeks post recruitment.

#### Mediterranean diet score

Analysis of covariance, adjusting for baseline MD score and using a significance level of 5%, will be used to compare the mean change in MD score from baseline to 12 and 24 weeks between the two groups. As this is a pilot study with a small sample size we will only be able to explore any indications of sustained adherence to the MD.

### Qualitative data analysis

Recorded, end-of-trial telephone interviews with a sample of participants will be transcribed verbatim and analysed for key emerging themes, using a thematic content analysis [28].

## Discussion

The development of this trial has followed the UK Medical Research Council’s framework for developing and evaluating complex interventions [29]. In the development phase, knowledge was used to formulate theory that informed a systematic review and meta-analysis [12]. The potential intervention was therefore modelled (that is, its process and outcomes). In the current stage – that is, feasibility and piloting – this exploratory trial has been developed to refine the practicality of the intervention (for example, to test procedures, to estimate recruitment and retention, to determine sample size). This may then lead to further piloting and feasibility work, or to a large-scale RCT.

This is an innovative pilot RCT to assess rates of recruitment and retention, and adherence to a MD pattern in the second and third trimesters of pregnancy. No RCT has yet investigated the role of the MD on allergy. To test the overarching hypothesis that a MD during pregnancy will reduce the risk of allergy in newborns, testable only in a larger RCT with follow-up of the infants born, a pilot trial of this nature is an essential stepping stone; given that the available epidemiological evidence supports a link between a MD and the prevention of allergy, the trial in which we are engaged is the next step in investigating a dietary intervention. Such an intervention has the potential to offer a brief, effective and cost-effective intervention at a key stage in the lifespan, which may reduce the allergy burden on individuals and on the NHS.

### Reporting and dissemination

Reporting will adhere to CONSORT [30]. A final report will be submitted to the funding body (Chief Scientist Office). Papers will be prepared for publication in peer-reviewed journals. Articles reporting the results will be circulated to all collaborators.

### Trial governance

In accordance with Good Clinical Practice Guidelines [31] and NHS research governance requirements, a Project Management Committee has been formed, which commented on proposed changes to the protocol and is monitoring the trial. An independent academic is overseeing any trial steering and data-monitoring and ethics matters that may arise.

### Trial timeline

The trial started 1 May 2012. Invitations/recruitment material sent to potential participants (pregnant women telephoning a centralised booking system to arrange a dating scan appointment). Participants randomised to the intervention arm or control arm at dating scan appointments was ongoing from June to December 2012. Start of 12-week follow-up is ongoing from September 2012. Start of 24 week follow-up is ongoing from December 2012. Planned trial end date is end July 2013. Duration of trial is 15 months.

## Trial status

At the time of submission, 22 participants had been recruited. At the end of recruitment (December 2012), 30 participants had been recruited.

## Abbreviations

FFQ: Food frequency questionnaire; MD: Mediterranean diet; NHS: National Health Service; RCT: Randomised controlled trial

## Competing interests

The authors declare that they have no competing interests.

## Authors’ contributions

DAS is the chief investigator and along with ASh conceived of the trial, designed the protocol and obtained the funding for the trial. VSH is the research fellow who is running the trial, and helped further develop the protocol. DAS, VSH and ASh produced the first draft of the paper, and AW (qualitative researcher), GD (clinician), AR (psychologist), CW (statistician) and ASt (health economist) sit on the Project Management Committee and contributed to the manuscript. All authors read and approved the final manuscript.
